# Dr. Tobias John Green

**Published:** 1916-12

**Authors:** 


**Dr. Tobias John Green,** Geneva 1845, died at his home in Mexico, N. Y., Oct. 4, aged 98. The death of these college mates, so nearly of an age, within a week reminds us that the history of this medical college ought to be written before its alumni have all passed away.

